# External factors affecting fertility, and how to correct their impact

**Published:** 2017-12

**Authors:** FH Comhaire, W Vandenberghe, WAE Decleer

**Affiliations:** Fertility Clinic, Weststraat 16-18, 9880 Aalter, Belgium; Lab Protein Science, Proteomics and Epigenetic Signaling, Dept Biomedical Sciences University Antwerp, Campus Drie Eiken, Universiteitsplein 1, 2610 Wilrijk, Belgium; IVF Center, AZ Jan Palfijn Gent, Watersportlaan 5, 9000 Gent, Belgium

**Keywords:** 8-OH-2 deoxyguanosine, epigenetics, hTERT, oxidative stress, infertility, food supplement

## Abstract

Fertility of both men and women has been negatively influenced by external factors and life style in recent decennia. Mechanisms of hormone disruption, oxidative damage, and epigenetic DNA changes play a pivotal role in this process.

In Belgium, strict regulations have been imposed to reduce the exposure to xeno-estrogens, which has resulted in a partial recovery of sperm quality. At the other hand, more couples require in vitro fertilisation (IVF) whereby ovarian stimulation may be associated with epigenetic DNA hyper-methylation of follicular cells, and increased risk of carcinogenesis among offspring.

In order to reduce the health risks for the offspring it is recommended to optimize the oxidative, epigenetic and metabolic situation of both parents by means of lifestyle adaptation, and the use of appropriate food supplementation before conception and during pregnancy.

## Introduction

The person’s genome is fixed at the time of conception, but it can be modified during life through the influence of external factors. Among the latter oxidative changes to the desoxyribonucleic acid (DNA), resulting in transition mutagenesis (Valavanidis et al., 2009), and epigenetic changes due to altered methylation and acetylation (Weinhold, 2006) are the most important. Epigenetics determine which fractions of the DNA come to expression and may promote the occurrence of particular diseases such as neurological disorders, diabetes, atheromatosis and cancer.

Changes in the structure and expression of DNA are pivotal during embryogenesis and may be influenced by, amongst other factors, life style, environmental toxins and nutrition (Torano et al., 2016). Since these changes are reversible they should be amenable for correction (Lee, 2015), as argued for in the present paper.

## Oxidative damage to spermatozoa

**Figure 1:**
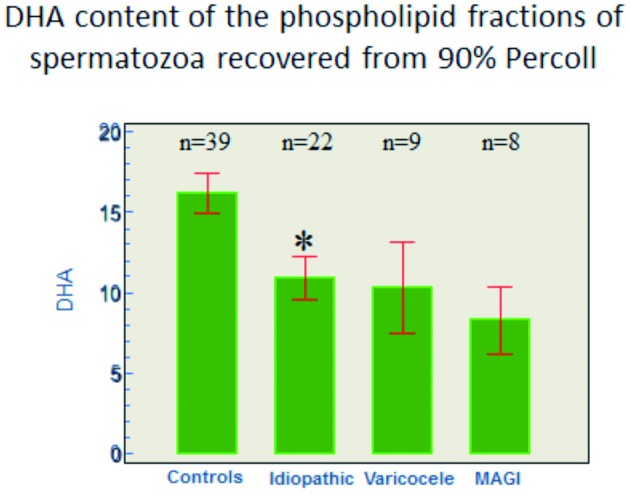
— The histogram shows the proportion of docosahexaenoic acid (DHA, or cervonic acid, on the vertical axis, in mole%, mean +/- SEM) in the phospholipid fractions of the membrane of spermatozoa of normal fertile men (controls) and of patients in whom infertility is due to idiopathic oligozoospermia, or to varicocele, or to male accessory gland infection (MAGI).

Male infertility is commonly caused by varicocele, accessory gland infection (prostato-epididymitis), or hormone disruption by environmental xeno- estrogens. In all these circumstances spermatozoa are subject to increased oxidative stress with an increased concentration of reactive oxygen species (ROS) in the ejaculate (Depuydt et al., 1996; Aitken et al., 1996; Aitken and Sawyer, 2003). These reduce the number of double bonds of the poly unsaturated fatty acids of the phospholipid layer that constitutes the sperm membrane. The proportion of the docosahexaenoic acid (22-6 ω3; cervonic acid) is decreased (Fig.1) reducing the fluidity of the membrane and its capacity to fuse with the oocyte membrane during fertilisation (Zalata et al., 1998).

ROS also affects the energy production by the mitochondria situated at the mid-piece which results in poor sperm motility (asthenozoospermia) (Comhaire et al., 1989; Aitken et al., 2016).

**Figure 2:**
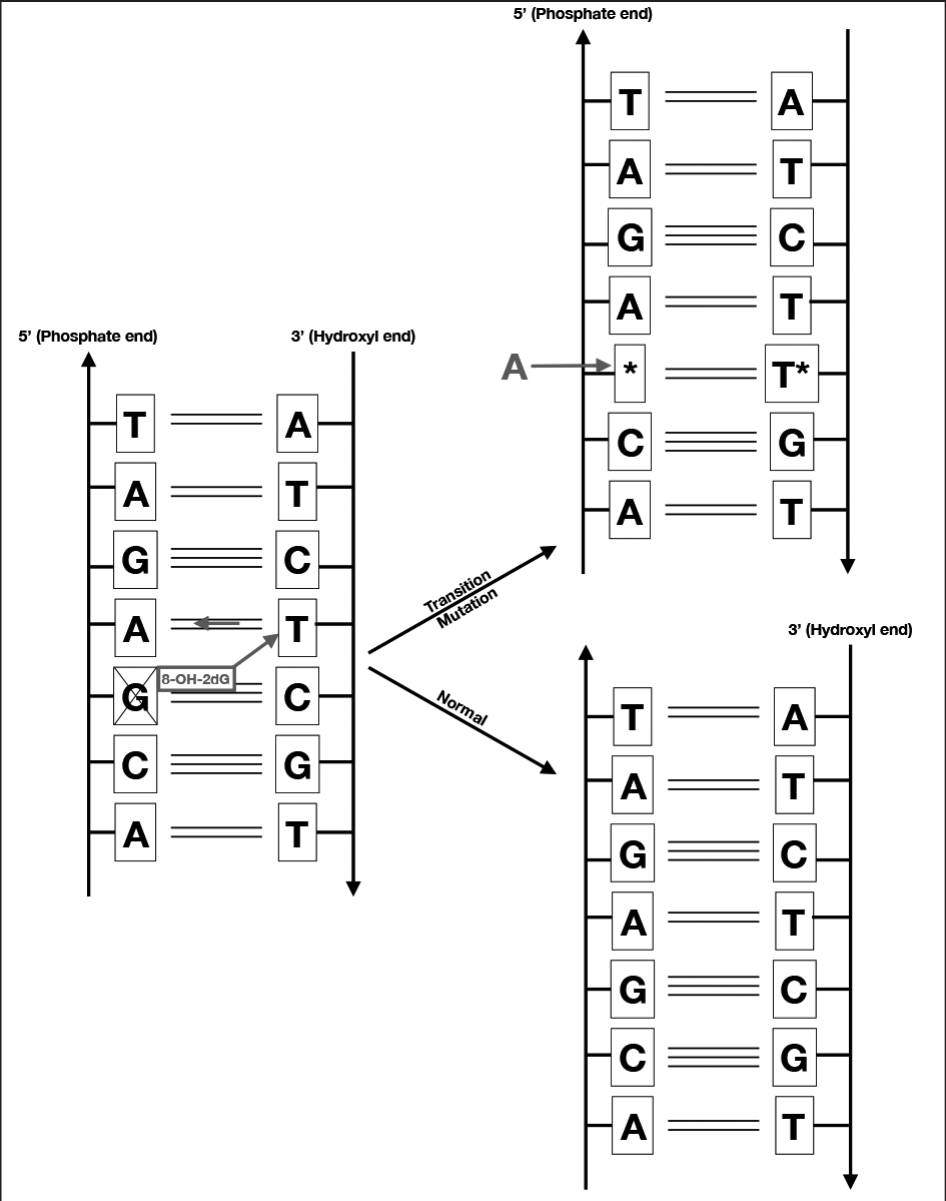
— The diagram shows the mechanism of transition mutagenesis occurring in oxidative DNA damage. Guanine (G) is oxidised into 8-hydroxy-2 deoxyguanosine (8-OH-2dG) by the effect of reactive oxygen species, or free radicals. During DNA replication the 8-OH-2dG will bind to Thymine (T), whereas it should normally bind to Cytosine (C). The dinucleotide Thymine- Adenine (A) will replace the dinucleotide G-C in the daughter cells, which constitutes a transition mutation.

Oxygen damage at the level of DNA converts the nucleotide guanine into 8-hydroxy-2-deoxy guanosine (8-OH-2dG), the amount of which is found to be increased in spermatozoa of infertile men (Comhaire et al., 2000). During cell replication after fertilisation, the 8-OH-2dG binds to thymine, whereas guanine would normally bind to cytosine, introducing transition mutagenesis in the daughter cells (Fig.2).

During normal conception or after intra uterine insemination or regular IVF, fusion of the sperm and oocytes membranes does not occur since the sperm membrane has been damaged by oxidative damage. Therefore, the altered DNA will not enter de oocytes. In case of ICSI, however, an excessive amount of oxidised DNA in the sperm heads may be introduced into the oocyte cytoplasm, exceeding the repair capacity of the P53 protein, and allowing for transition mutagenesis of the embryos. This may result in early abortion or impaired neurological or psychological development of the offspring (Aitken et al., 2016).

Reactive oxygen species may also induce DNA strand breaks which can be demonstrated and quantified by determining the DNA fragmentation index (DFI). The use of sperm with an elevated proportion of DFI is associated with a lower success rate in intrauterine insemination, where IVF with ICSI should be the method of choice (Giwercman et al., 2010)

## Oxidative damage to oocytes

Common causes of female infertility, namely disturbed ovulation e.g. by the polycystic ovary syndrome, pelvic inflammatory disease, or endometriosis are associated with inflammation inducing increased concentrations of cytokines, prostaglandins and ROS (Da Broi et al. 2018). These will influence the follicular cells and may damage both the membrane and the DNA of the oocytes, and reduce the probability of fertilisation (Agarwal et al., 2005). There is evidence that epigenetic changes of oocyte DNA may also occur due to the intercellular exchange of signals between the cells of the corona radiata and the oocytes through gap junctions (Downs, 2001; Fair, 2003).

## Oxidative damage during embryogenesis

After fertilisation the oocyte divides into daughter cells whereby forces are exerted to fold the membrane, and energy is needed to replicate the DNA. The former is obtained by the contraction of a specific protein, the non-muscle myosin II (Conti and Adelstein, 2008). The energy that is required for these processes is provided as adenosine triphosphate generated by the mitochondria. Inhibition of mitochondrial function by inflammation and ROS may reduce energy production (Michael et al., 2006), causing inadequate contraction of the myosin proteins and impaired embryonic cell division.

## Epigenetic changes during ovarian stimulation

Recent research suggests that the risk of certain cancers and of leukaemia may be increased among children born after IVF (Källén et al., 2010; Petridou et al., 2012). In another study such increased risk was not confirmed except for hepatoblastoma and rhabdomyosarcoma (Williams et al., 2013). During ovarian stimulation certain biochemical changes do occur in follicular fluid which could affect epigenetics, DNA methylation in particular. In fact, the concentration of homocysteine is commonly increased, deregulating the balance between S-adenosyl methionine (SAMe) and S-adenyl homocysteine (SAH) of the one carbon metabolism (Boxmeer et al., 2008; Ocal et al., 2012). This may induce increased activity of DNA methyltransferase in the granulosa cells and the oocytes, via the intercellular gap-junctions.

**Figure 3:**
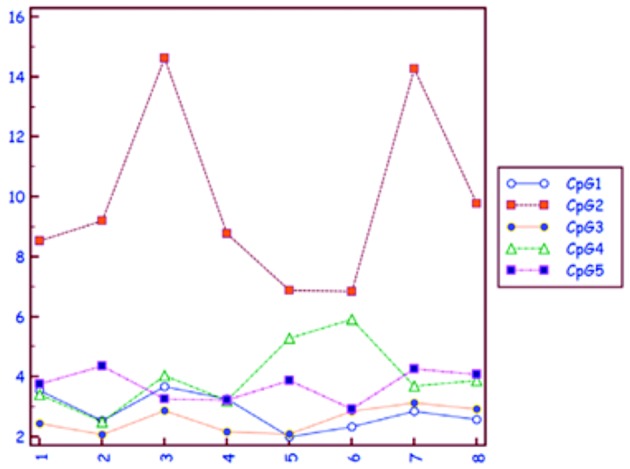
— In this line and dot diagram the proportion of methylation (on the vertical axis, in %) of each one of the 5 dinucleotides of the promoter region of the human telomerase reverse transcriptase (hTERT) is represented. The level of methylation of the Cytosine-Guanine 2 (CpG2) reaches 15% in two out of eight samples (samples number 3 and 7, on the horizontal axis) of follicular fluid of patients treated by IVF with ovarian stimulation.

We have measured the degree of methylation of the human telomerase reverse transcriptase (hTERT) in follicular fluid cells of 8 ovaries obtained during follicular aspiration for IVF. Using pyrosequencing we could ascertain enhanced methylation of the second CpG dinucleotiden of the hTERT promoter region in 2 out of 8 samples (Comhaire et al., 2015) (Fig.3).

The elevated methylation of the dinucleotides results in suppression of the catabolic subunit of the telomerase. The anabolic telomerase subunit becomes unopposed and lengthens the telomeres (Guilleret et al., 2002), which has been associated with increased risk of childhood brain tumours and of hepatocellular hepatocarcinoma (Castelo-Branco et al., 2013; Zhang et al., 2005).

## Hypothesis

In summary, it appears that ovarian stimulation deregulates the balance between S-adenosyl methionine and S-adenosyl homocysteine, enhancing the activity of DNA-methyltransferase through the one carbon metabolism. This may induce hyper-methylation of the second GpG dinucleotide of the hTERT promoter region and increase telomere length of the cumulus cells, which signal is transferred to the oocytes. Together with DNA changes introduced by oxidative damage and inflammatory cytokines, this mechanism may increase the risk of abnormal cell division, of impaired DNA replication, and of excessive telomere length interfering with the development and health of the offspring.

## Prevention of DNA impairment

Since oxidative, inflammatory and epigenetic alterations may cause health problems in offspring of infertile couples, particularly if ovarian stimulation is applied, it is important to optimise the biological internal environment (“milieu intérieur”) of both partners before and during conception, and during pregnancy. Evidently, recommendations should include prohibition to smoke and to drink alcohol, as well as a well-balanced diet. In addition it is suggested to prescribe a specific food supplement (Arhin et al., 2017) composed of certain vitamins (DeVilbiss et al., 2017), minerals, amino acids, and plant extracts. Such supplements are called nutriceuticals, and they aim at decreasing oxidative overload and inflammation, at optimising mitochondrial function, and at reducing epigenetic alterations.

**Figure 4:**
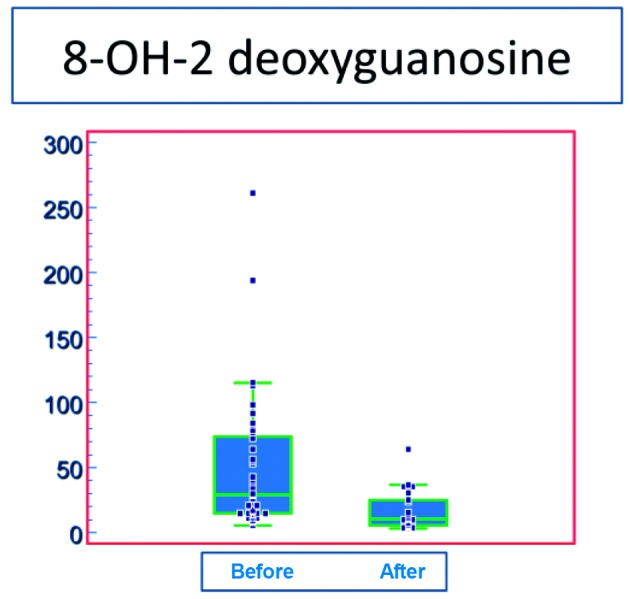
— This box and whisker plots shows the concentration of 8-OH-2dG (on the vertical axis, in fMol) in the DNA of spermatozoa of infertile men, before treatment and after 6 weeks of intake of the anti-oxidant Astaxanthine (8 mg per day).

The formulation which we have created is composed of the membrane antioxidant Astaxanthin, which is a non-toxic carotenoid present in the biomass of the alga Haematococcus pluvialis (Comhaire and Mahmoud, 2003; Comhaire et al., 2005), and the mitochondrial antioxidant oxidoreductase Ubiquinone Q10 (Garrido-Maraver et al., 2014; Showell et al., 2014; Sekhon et al., 2010). Astaxanthin was proven to reduce the amount of oxidised DNA in the spermatozoa of infertile men (Fig.4).

The extract of the Mediterranean pine bark (Pinus maritima) contains proanthocyanidins with both anti-oxidant and anti-inflammatory effect through the inhibition of the cyclo-oxygenase 2 (COX- 2) and the 5-lipoxygenase (5-LOX) (Canali et al., 2009). ATP production by the mitochondria is optimized by stimulating the beta-oxidase activity by the addition of the poly-unsaturated omega-3 fatty acids together with l-acetyl-carnitine (Lenzi et al., 2004). The combination of the vitamins B6, B9 and B12 reduces the concentration of homocysteine concentration restoring the balance between S-adenosyl-methionine (SAMe) and S-adenosyl- homocystein (SAH) which should normalise the degree of DNA-methylation (Homocysteine Lowering Trialists’ Collaboration, 2005; Hoyo et al., 2011; McCullough et al., 2016). This effect is reinforced by the addition of selenomethionine (Speckmann and Grune, 2015).

A limited randomised prospective double-blind placebo-controlled trial (RCT) has evidenced an increased rate of ongoing pregnancies after IVF among couple who were pre-treated by the nutriceutical, with number needed (NNT) to treat equal to 4 (Comhaire and Mahmoud, 2013). Melatonin has been shown to exhibit unique oxygen scavenging abilities. Some studies have suggested a role for melatonin in gamete biology. Clinical studies suggest that melatonin supplementation in IVF may lead to better pregnancy rates. This, however, needs confirmation by future well designed clinical trials (Fernando and Rombauts, 2014)

## Conclusion

There is conclusive evidence to sustain the concept of external factors negatively influencing the fertilizing potential of human gametes as well as potentially disturbing the embryo development, particularly after ovarian stimulation. These may explain, at least in part, the failures of ongoing pregnancies after IVF, and the alleged increase of certain malignant diseases and neurological disorders observed in offspring of fertility treatments. It is mandatory to create an optimal “milieu intérieur” in both partners before conception and during pregnancy, for which nutritional supplementation may be recommended.
